# Family Functioning and Adolescent Internalizing and Externalizing Problems: Disentangling between-, and Within-Family Associations

**DOI:** 10.1007/s10964-019-01094-z

**Published:** 2019-08-05

**Authors:** Stefanos Mastrotheodoros, Catarina Canário, Maria Cristina Gugliandolo, Marina Merkas, Loes Keijsers

**Affiliations:** 1 Research Center Adolescent Development, Utrecht University, Utrecht, The Netherlands; 2 Department of Psychology, University of Athens, Athens, Greece; 3 Faculty of Psychology and Education Science of the University of Porto, Porto, Portugal; 4 Department of Human, Social and Health Sciences, University of Cassino and South Latium, Cassino, Italy; 5 Department of Psychology, Catholic University of Croatia, Zagreb, Croatia; 6 Department Developmental Psychology, TSB, Tilburg University, Tilburg, The Netherlands

**Keywords:** Adolescence, Family functioning, Internalizing, Externalizing, Random-intercept cross-lagged panel models, Within-family

## Abstract

Adolescence is often a period of onset for internalizing and externalizing problems. At the same time, adolescent maturation and increasing autonomy from parents push for changes in family functioning. Even though theoretically expected links among the changes in family functioning and adolescent internalizing and externalizing problems exist, studies examining this link on the within-family level are lacking. This longitudinal, pre-registered, and open-science study, examined the within-family dynamic longitudinal associations among family functioning, and internalizing and externalizing problems. Greek adolescents (*N* = 480, *M*_age_ = 15.73, 47.9% girls, at Wave 1) completed self-report questionnaires, three times in 12 months. Random-Intercept Cross-Lagged Panel Models (RI-CLPM) were applied; such models explicitly disentangle between-family differences from within-family processes, thereby offering a more stringent examination of within-family hypotheses. Results showed that family functioning was not significantly associated with internalizing or externalizing problems, on the within-family level. Also, alternative standard Cross-Lagged Panel Models (CLPM) were applied; such models have been recently criticized for failing to explicitly disentangle between-family variance from within-family variance, but they have been the standard approach to investigating questions of temporal ordering. Results from these analyses offered evidence that adolescents with higher internalizing and externalizing problems compared to their peers, tended to be those who later experienced worse family functioning, but not vice versa. Implications for theory and practice are discussed.

## Introduction

Adolescence is a period of vast changes on multiple levels (cognitive, emotional, social). Adjusting to these changes can be challenging for adolescents, who often experience an increase in internalizing (Graber 2013) and externalizing problems (Georgiou and Symeou 2018). At the same time, adolescents’ families need to adapt to the adolescent’s increasing needs for autonomy and independence, something that may lead to a temporary decrease in positive family functioning (De Goede et al. 2009). Theoretical accounts of adolescent development posit that youth develop in multiple contexts (Bronfenbrenner and Morris 2006), of which families are the most proximal and influential. Thus, developmental perspectives postulate that changes in family functioning will trigger changes in adolescent internalizing and externalizing problems. At the same time, multiple theoretical accounts highlight that this influence might be bidirectional (Crouter and Booth 2003). Family systems theories (Cox et al. 2001), such as the Circumplex Model of Marital and Family Systems (Olson 2000), posit that the family system consists of sub-systems, which dynamically affect each other. Therefore, based on family systems perspectives, adolescent adaptation (as seen in the presence or absence of internalizing and externalizing problems) may also affect other subsystems that define family functioning, such as the parent-child relationship, as well as the family as a whole.

Although there is an increasing number of studies addressing how adolescent adaptation is reciprocally linked to parenting (Keijsers et al. 2011), and how parenting changes during adolescence (Mastrotheodoros et al. 2018), only a few studies have examined the bidirectional links between family functioning (at the system level) and adolescent adaptation. Furthermore, even though the dynamic processes are theoretically postulated on the within-family level, almost no studies on family functioning have employed techniques that adequately disentangle the within-family effects (Keijsers 2016), from the between-family differences and associations (Hamaker et al. 2015). The current pre-registered longitudinal study investigated the direction of the longitudinal effects between family functioning and adolescent internalizing and externalizing problems, while taking into account the disaggregation of the between-person differences from the within-person effects, using Random Intercept Cross-Lagged Panel Models.

### Family Functioning and Adolescent Internalizing and Externalizing Problems

Although extant research has found evidence for the link between adolescent adaptation and parenting, the family systems approach states that there are qualities of the system as a whole that predict adolescent adaptation above and beyond the dyadic relationship qualities. The Circumplex Model of Marital and Family Systems (Olson 2000) is a prominent theoretical framework, which emphasizes three important family system qualities: family flexibility, cohesion, and communication. Flexibility describes the quality and expression of leadership and organization, roles, and rules in the family. Cohesion describes the emotional bonding among family members. Communication describes the degree to which members openly discuss and express their views, and needs (Olson 2011). These dimensions have been extensively used to investigate family functioning (e.g., Olson 2011), as seen in different measurement instruments, and family therapy approaches (Walsh 2003). Thus, to assess associations between family functioning and adolescents’ internalizing and externalizing problems, it is important to measure and investigate how the family functions as a system (e.g., Delsing et al. 2005).

Family relationships transform during adolescence (Mastrotheodoros et al. 2019a), and this transformation might be stressful for adolescents and the family system as a whole. During adolescence, a good fit between the adolescent’s developmental needs (e.g., for autonomy), and the opportunities offered by the environment (e.g., the family) are expected to facilitate adolescent adaptation (Gutman and Eccles 2007). Therefore, it is expected that cohesive, flexible, and openly communicating families will accommodate adolescents’ needs without major disruptions. Conversely, families that are distant, inflexible, and do not facilitate open communication, may constitute a misfit for adolescent development, which is expected to cause an increase in the stress levels of adolescents (see stage-environment fit hypothesis, Gutman and Eccles 2007), who already struggle to transition from childhood to young adulthood.

Unlike the vast majority of studies on the link of adolescent adaptation with parenting (e.g., Keijsers et al. 2011), empirical evidence on the association between dimensions of family functioning and internalizing and externalizing problems in adolescence is relatively scarce. Family functioning, as well as a positive family climate, have been negatively linked to adolescents’ depressive symptoms over time (Klasen et al. 2015). Also, family flexibility was found to be negatively related to adolescents’ depressive symptoms, internalizing and externalizing problems (Joh et al. 2013), and has been identified as a better predictor than family cohesion for identifying at-risk behaviors (Tafà and Baiocco 2009). Family cohesion was found to be negatively related to adolescents’ depressive symptoms, but not related to anxiety symptoms (White et al. 2014). Furthermore, family communication was negatively related to internalizing and externalizing behaviors (Elgar et al. 2013) and was shown to be effective in reducing conduct problems (Molleda et al. 2017).

Most likely, such influences are reciprocal in nature, running not only from the family to the adolescent, but also vice versa (Crocetti et al. 2016). Thus, adolescents’ internalizing and externalizing problems can also have an effect on how the family functions as a whole. In fact, empirical work suggests that family flexibility, cohesion, and communication may decrease in families with a seriously mentally ill person (Koutra et al. 2014), but how sub-clinical internalizing and externalizing problems during adolescence affect family functioning is not yet clarified. More specifically, studies on the relation between family functioning and adolescents’ psychological outcomes are mostly cross-sectional in nature (Queen et al. 2013). Even though few recent studies used longitudinal designs, they did not investigate the direction of effects (e.g., White et al. 2014). Given the dearth of relevant empirical evidence, research should address the directionality of the relation between family functioning and adolescents’ psychological outcomes using longitudinal, cross-lagged designs.

The theoretical ideas regarding how adolescent adaptation and family functioning are reciprocally related pertain to processes within families. Thus, changes in the adolescent and the family system of the same family are related. However, hardly any study has been conducted on the within-family level (Hamaker et al. 2015; Keijsers and van Roekel 2018), in what regards empirical research of family effects and the reciprocity therein. Almost all studies on the topic of family functioning and adolescent internalizing and externalizing problems have focused on between-family differences or associations, by comparing families with each other, for instance with correlational designs (for an exception, see Kapetanovic et al. 2019). Some recent empirical studies highlight how both ecological levels, the between-family and the within-family level, might yield different insights, for instance regarding negotiations of privacy boundaries with parents (Dietvorst et al. 2018), or the spillover of interparental conflict on parenting (Mastrotheodoros et al. 2019b). In fact, in some situations, the within-, and between-family effects may be opposite in sign (Dietvorst et al. 2018), a situation called a Simpson’s paradox (Kievit et al. 2013).

## Current Study

Family systems perspectives propose that family functioning is influential for adolescent adaptation (e.g., Cox et al. 2001). They suggest that if family functioning deteriorates, this will negatively impact adolescent internalizing and externalizing symptoms. An important aspect of this hypothesis is that it focuses within one family, hence, on the within-family level. Even though extant research has improved our understanding of how family functioning and parenting dynamically associate with adolescent adaptation (Crocetti et al. 2016), a focus on within-family processes while controlling for between-family stable differences is a more appropriate test of the theory (e.g., Hamaker et al. 2015; Keijsers 2016), and holds the potential to further expand our understanding of adolescent development (Keijsers and van Roekel 2018).

To test the theoretical premises on the correct ecological level of inference, the present study investigated to what extent dynamic associations between family functioning and internalizing and externalizing problems were present at the within-family level, by applying Random Intercept Cross-Lagged Panel Models (Hamaker et al. 2015; Keijsers 2016). These novel structural equation models are suitable for differentiating reciprocal associations at the within-family level, from stable associations at the between-family level. Based on the existing empirical evidence about the relationship between family functioning and adolescent internalizing and externalizing problems at the between-family level (e.g., Crocetti et al. 2016), and guided by the family systems (Cox et al. 2001) and family developmental theoretical perspectives (e.g., Georgiou and Symeou 2018), it was hypothesized that family cohesion, flexibility, and communication will have a significant longitudinal negative within-family effect on internalizing and externalizing problems (Hypothesis 1). Thus, it was expected that periods with decreased cohesion, flexibility and communication would precede periods with heightened internalizing and externalizing problems. Additionally, it was hypothesized that internalizing and externalizing problems will have longitudinal negative within-person effects on family cohesion, flexibility and communication (Hypothesis 2).

## Method

### Sample

The sample for this study consisted of 480 Greek adolescent students (47.9% girls, 15.7 years old at T1) attending 8 high schools in Athens, Greece. The schools were selected from the pool of all the high schools in Attiki (the prefecture Athens lies in, with more than a third of the country’s population). Access to this pool was given by the Greek Ministry of Education. In order to broaden the population of interest, from this pool 8 high-schools from different parts of Athens metropolitan area were selected. These parts corresponded to different socio-economic strata: 3 schools from the center of Athens (low/lower-middle class), 3 schools from the western, southern, and eastern parts of the city (middle class areas), one school from the northern suburbs (upper-middle class), and one school from a less-urbanized town on the east of Athens (middle class).

The students were assessed three times in 12 months (two 6-month intervals), between March 2012 and March 2013. The procedures were identical in all three waves. Trained assistant researchers along with the first author visited the classrooms during school hours. Questionnaire completion took part in 2-hour slots, after the school principal’s permission.

### Measures

#### Family functioning

To assess the dimensions of family functioning, the Family Adaptability and Cohesion Evaluation Scales—IV (FACES-IV, Olson 2011) was used. The FACES-IV has been translated and adapted in Greek, and it has shown good psychometric properties in Greek samples (Koutra et al. 2013).

#### Flexibility

The Balanced Flexibility subscale from the FACES—IV (Olson 2011) was used to assess family flexibility. This scale consists of 7 items that are addressed in a likert scale that ranges from 1 (*Strongly Disagree*) to 5 (*Strongly Agree*). Example items are: “Our family tries new ways of dealing with problems” and “My family is able to adjust to change when necessary”. In the current study, this scale showed satisfactory internal consistency ranging from *α* = 0.66 to *α* = 0.69 from Wave 1 to Wave 3. The Intraclass Correlation Coefficient (ICC) was 0.58, indicating that 58% of the variance was due to stable differences between-families and the remainder 42% was due to fluctuations over time, or variance within-families.

#### Cohesion

The Balanced Cohesion subscale from the FACES-IV (Olson 2011) was used to assess family cohesion. This scale consists of 7 items that are addressed in a likert scale that ranges from 1 (*Strongly Disagree*) to 5 (*Strongly Agree*). Example items are: “Family members feel very close to each other”, and “Family members are supportive of each other during difficult times”. In the current study, this scale showed satisfactory internal consistency ranging from *α* = 0.71 to *α* = 0.75 from Wave 1 to Wave 3. The ICC was 0.61, indicating that 39% of the variance was within-family variance.

#### Communication

The Family Communication scale from the FACES-IV (Olson 2011) was used to assess family communication. This scale is based on the Parent-Adolescent Communication scale (Barnes and Olson 1985) which was revised and included in the 4th edition of the FACES. This scale consists of 10 items that are addressed in a likert scale that ranges from 1 (*Strongly Disagree*) to 5 (*Strongly Agree*). Example items are: “Family members are satisfied with how they communicate with each other”, and “Family members can calmly discuss problems with each other”. In the current study, this scale showed good internal consistency ranging from *α* = 0.89 to *α* = 0.90 from Wave 1 to Wave 3. With an ICC of 0.62, 38% of the variance was allocated at the within-family level.

### Internalizing and Externalizing Problems

#### Depressive Symptoms

The Greek version of the Symptoms Checklist 90—Revised (SCL-90R, Donias et al. 1991) was used to measure symptoms of depression. The Depression subscale consists of 13 items which are addressed on a 5-point likert scale, from 0 (*Not at all*) to 4 (*Very much*). An example item is “How much were you bothered by feeling low on energy or slowed down?”. In the current study, the scale showed good internal consistency, with Cronbach’s *α* ranging from 0.83 to 0.88, from Wave 1 to Wave 3. The ICC was 0.66, indicating that 34% of the variance was due to within-person fluctuations over time.

#### Anxiety

The Greek version of the SCL-90R (Donias et al. 1991) was used to measure symptoms of anxiety. The Anxiety subscale consists of 10 items that are addressed on a 5-point likert scale, from 0 (*Not at all*) to 4 (*Very much*). An example item is “How much were you bothered by nervousness or shakiness inside?”. The internal consistency in the current study was good, with Cronbach’s *α* ranging from 0.79 to 0.84, from Wave 1 to Wave 3. The ICC was 0.64. Thus, 36% of the variance was within-person variance.

#### Anger

The Anger subscale from the Greek SCL-90R (Donias et al. 1991) was used to measure anger. This subscale consists of 6 items that are addressed on a 5-point likert scale, from 0 (*Not at all*) to 4 (*Very much*). An example item is “How much were you bothered by outbursts of anger that you could not control?”. In this study, Cronbach’s *α* were good, ranging from 0.81 to 0.85, from Wave 1 to Wave 3. The ICC was 0.58, indicating that 42% of the variance was due to within-person fluctuations in anger.

### Preregistered Analytic Procedure

**Fig. 1:**
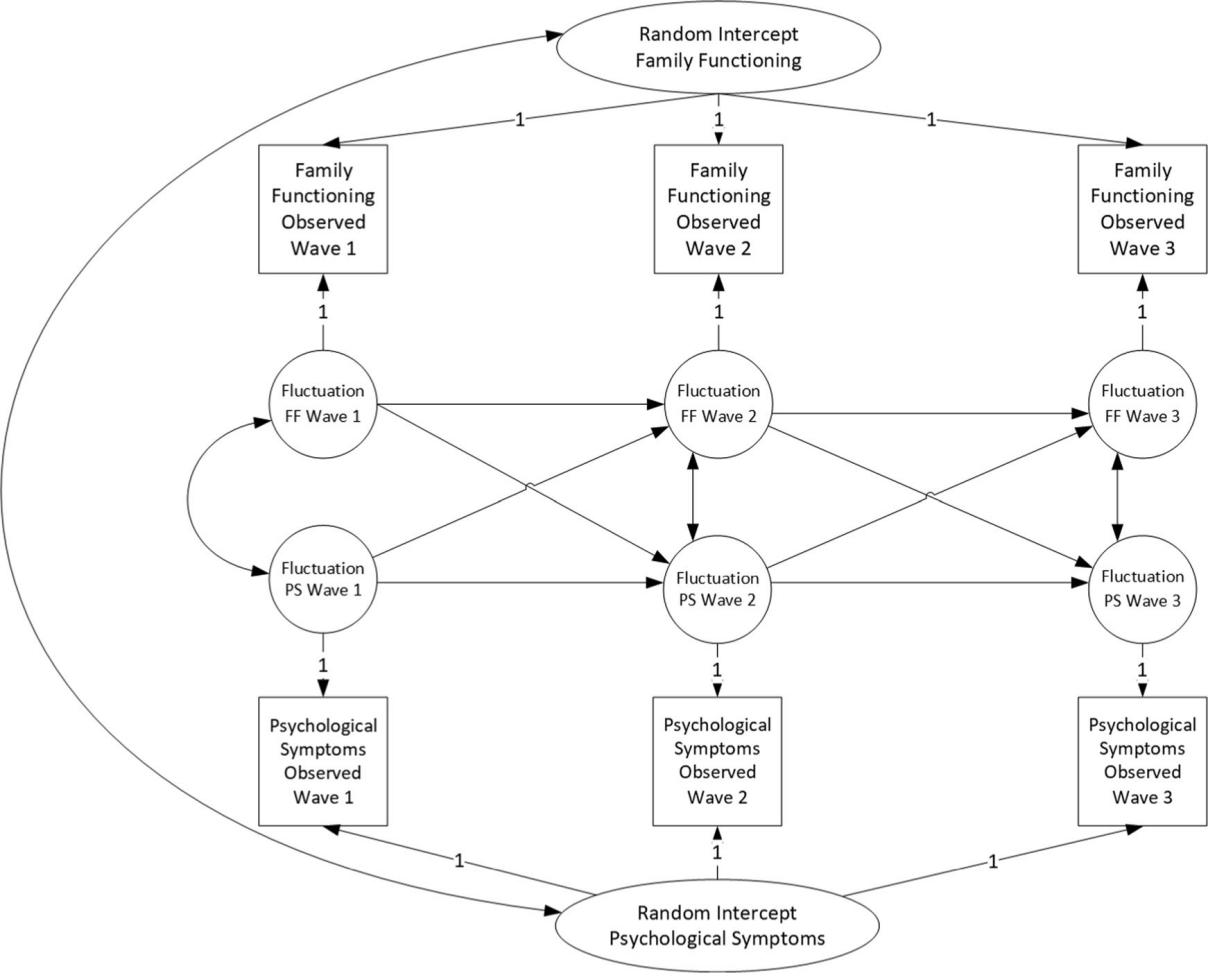
Random intercept cross-lagged panel model as applied in this study

Random-Intercept Cross-Lagged Panel Models (RICLPM, Hamaker et al. 2015) were applied to disaggregate within-, from between-family processes and answer the research questions. The analytic plan for this study has been pre-registered on the Open Science Framework on November 23rd, 2018 (anonymized link: https://osf.io/8f95w/?view_only=ef1f8f29824942889039bde2fa983994). The procedure described in detail in the pre-registered document, and briefly summarized here, was followed. First, as planned, data were screened for missing data. Little’s MCAR test was significant, but the normed chi-square (*χ*^*2*^/df) was low (386/279 = 1.38), implying a small violation of the MCAR assumption. Therefore, Full Information Maximum Likelihood, with Robust standard errors (MLR, Satorra and Bentler 2001) was applied. With respect to the ICC, there was more than 10% of variance on the within-person level for each measure. Thus, applying an analytic technique that explicitly disaggregates the two levels of variance was warranted. Nine bivariate RICLPMs were specified, per combination of family and outcome dimensions (Table 1). The basic model was a fully constrained model, with carry-over stability and cross-lagged effects constrained over time, as shown in Fig. 1. Finally, as planned, alternative models were applied, which consisted of different specifications of the RICLPM (i.e., an unconstrained RICLPM), as well as standard Cross-Lagged Panel Models.

**Table 1:** List of predictors and outcome measures

Family functioning dimensions	Adolescent outcomes
Family flexibility	Depressive symptoms
Family cohesion	Anxiety symptoms
Family communication	Aggression

## Results

### Descriptive Statistics

Table 2 presents the descriptive statistics (means, standard deviations, and Cronbach’s α) of the study variables.

**Table 2:** Descriptive statistics (means, standard deviations, and internal consistency coefficients *α*) for all study variables

	*M*	*SD*	*α*
Age	15.73	0.82	
Depressive symptoms Τ1	0.95	0.67	0.85
Depressive symptoms Τ2	0.92	0.64	0.83
Depressive symptoms Τ3	0.88	0.71	0.88
Anxiety Τ1	0.83	0.62	0.79
Anxiety Τ2	0.76	0.58	0.80
Anxiety Τ3	0.71	0.63	0.84
Anger Τ1	1.11	0.88	0.82
Anger Τ2	1.00	0.88	0.85
Anger Τ3	0.97	0.86	0.85
Flexibility Τ1	23.31	4.38	0.66
Flexibility Τ2	23.36	4.50	0.69
Flexibility Τ3	23.21	4.50	0.68
Cohesion Τ1	24.39	4.73	0.71
Cohesion Τ2	24.65	4.65	0.73
Cohesion Τ3	24.56	4.65	0.75
Communication Τ1	29.63	6.07	0.89
Communication Τ2	29.39	5.97	0.88
Communication Τ3	28.92	6.40	0.90

### Within-Person Dynamic Associations Among Family Functioning and Internalizing and Externalizing Problems

Table 3 presents the fit indices for the bivariate RICLPMs. All models had an acceptable fit (Table 3), according to the predetermined criteria of RMSEA < 0.08, CFI > 0.90 and TLI > 0.90. Tables 4–6 present the parameter estimates of the RICLPMs for each family functioning dimension with each of the outcome measures. In these models, four types of effects are provided: between-family correlations, within-time associations (correlated change), within-family stability effects, and within-family cross-lagged effects. The syntaxes can be found following this OSF link: https://osf.io/r9kpy/?view_only=0d55d50910274dd8acba47fa28738d98.

**Table 3:** Model fit indices for all models

	Model Type	*χ*^2^	*df*	CFI	TLI	RMSEA
Flexibility—depressive symptoms	RICLPM-fixed	14.433	5	0.988	0.964	0.063
Flexibility—depressive symptoms*	CLPM	16.214	7	0.988	0.975	0.052
Flexibility—depressive symptoms*	RICLPM-free	2.716	1	0.998	0.968	0.060
Flexibility—anxiety	RICLPM-fixed	17.601	5	0.984	0.951	0.073
Flexibility—anxiety	CLPM	20.939	7	0.982	0.962	0.064
Flexibility—anxiety*	RICLPM-free	2.304	1	0.998	0.975	0.052
Flexibility—anger	RICLPM-fixed	12.153	5	0.990	0.971	0.055
Flexibility—anger	CLPM	21.280	7	0.981	0.959	0.065
Flexibility—anger	RICLPM-free	4.786	1	0.995	0.924	0.089
Cohesion—depressive symptoms	RICLPM-fixed	17.407	5	0.985	0.954	0.072
Cohesion—depressive symptoms*	CLPM	13.878	7	0.991	0.982	0.045
Cohesion—depressive symptoms	RICLPM-free	6.688	1	0.993	0.894	0.109
Cohesion—anxiety	RICLPM-fixed	16.380	5	0.986	0.957	0.069
Cohesion—anxiety*	CLPM	14.152	7	0.991	0.981	0.046
Cohesion—anxiety*	RICLPM-free	5.366	1	0.994	0.917	0.095
Cohesion—anger	RICLPM-fixed	18.861	5	0.982	0.945	0.076
Cohesion—anger*	CLPM	12.016	7	0.993	0.986	0.039
Cohesion—anger	RICLPM-free	14.313	1	0.982	0.736	0.167
Communication—depressive symptoms	RICLPM-fixed	13.790	5	0.990	0.969	0.061
Communication—depressive symptoms*	CLPM	4.073	7	1.000	1.007	0.000
Communication—depressive symptoms	RICLPM-free	3.507	1	0.997	0.956	0.072
Communication—anxiety	RICLPM-fixed	18.596	5	0.984	0.951	0.075
Communication—anxiety*	CLPM	13.830	7	0.992	0.982	0.045
Communication—anxiety*	RICLPM-free	0.633	1	1.000	1.007	0.000
Communication—anger	RICLPM-fixed	13.720	5	0.989	0.967	0.060
Communication—anger*	CLPM	13.911	7	0.991	0.982	0.045
Communication—anger*	RICLPM-free	2.117	1	0.999	0.983	0.040

*RICLPM-fixed* random-intercept cross-lagged panel models with time invariance constrains on the autoregressive stabilities and the cross-lagged effects, *CLPM* cross lagged panel model, *RICLPM-free* fully unconstrained random intercept cross lagged panel model, *CFI* comparative fit index, *TLI* Tucker–Lewis Index, *RMSEA* root mean square error of approximation

*This model has better fit than the initial/original model, and therefore alternative model results are presented in the Supplementary Material

**Table 4:** Parameter estimates for the bivariate fixed RICLPMs modelling family flexibility with depressive symptoms, anxiety, and anger

Family Flexibility	Depressive symptoms				Anxiety				Anger			
	B	SE	*p*	*β*	B	SE	*p*	*β*	B	SE	*p*	*β*
Correlations												
Between-Person	−0.299	0.129	0.021	−0.149	−0.327	0.148	0.027	−0.186	−0.322	0.212	0.129	−0.149
T1	−0.112	0.081	0.168	−0.096	−0.151	0.090	0.091	−0.131	−0.285	0.164	0.083	−0.166
Cross-lagged effects												
Problems 1 → Flex. 2	0.028	0.636	0.965	0.005	0.409	0.811	0.614	0.068	−0.259	0.611	0.671	−0.062
Problems 2 → Flex. 3	0.028	0.636	0.965	0.003	0.409	0.811	0.614	0.045	−0.259	0.611	0.671	−0.062
Flex. 1 → Problems 2	0.011	0.013	0.413	0.107	0.004	0.013	0.752	0.036	−0.004	0.023	0.862	−0.017
Flex. 2 → Problems 3	0.011	0.013	0.413	0.064	0.004	0.013	0.752	0.028	−0.004	0.023	0.862	−0.019
Stability paths												
Flex. 1 → Flex. 2	−0.144	0.133	0.282	−0.148	−0.159	0.135	0.236	−0.164	−0.128	0.163	0.433	−0.127
Flex. 2 → Flex. 3	−0.144	0.133	0.282	−0.137	−0.159	0.135	0.236	−0.151	−0.128	0.163	0.433	−0.126
Problems 1 → Problems 2	−0.166	0.105	0.115	−0.269	0.028	0.158	0.860	0.040	0.300	0.192	0.118	0.295
Problems 2 → Problems 3	−0.166	0.105	0.115	−0.103	0.028	0.158	0.860	0.022	0.300	0.192	0.118	0.341
Correlated change												
T2	0.154	0.173	0.374	0.235	0.079	0.180	0.660	0.104	−0.236	0.341	0.490	−0.141
T3	0.023	0.097	0.815	0.020	−0.019	0.087	0.828	−0.019	−0.054	0.142	0.703	−0.037

Problems: denotes the internalizing and externalizing problems, as specified in the columns; Flex.: family flexibility

**Table 5:** Parameter estimates for the bivariate fixed RICLPMs modelling family cohesion with depressive symptoms, anxiety, and anger

Family Cohesion	Depressive symptoms				Anxiety				Anger			
	B	SE	*p*	*β*	B	SE	*p*	*β*	B	SE	*p*	*β*
Correlations												
Between-Person	−0.287	0.167	0.086	−0.143	−0.117	0.206	0.571	−0.076	−0.314	0.271	0.246	−0.149
T1	−0.112	0.110	0.307	−0.080	−0.224	0.134	0.095	−0.154	−0.117	0.262	0.656	−0.056
Cross-Lagged Effects												
Problems 1 → Cohesion 2	−0.241	0.781	0.758	−0.035	−0.189	0.870	0.172	−0.170	−0.547	0.589	0.353	−0.119
Problems 2 → Cohesion 3	−0.241	0.781	0.758	−0.022	−0.189	0.870	0.172	−0.164	−0.547	0.589	0.353	−0.123
Cohesion 1 → Problems 2	−0.006	0.014	0.653	−0.077	−0.021	0.015	0.167	−0.159	−0.010	0.025	0.706	−0.045
Cohesion 2 → Problems 3	−0.006	0.014	0.653	−0.046	−0.021	0.015	0.167	−0.159	−0.010	0.025	0.706	−0.048
Stability Paths												
Cohesion 1 → Cohesion 2	0.141	0.128	0.270	0.151	0.155	0.120	0.195	0.157	0.126	0.139	0.363	0.135
Cohesion 2 → Cohesion 3	0.141	0.128	0.270	0.141	0.155	0.120	0.195	0.158	0.126	0.139	0.363	0.126
Problems 1 → Problems 2	−0.166	0.114	0.144	−0.266	0.301	0.477	0.528	0.319	0.335	0.189	0.076	0.325
Problems 2 → Problems 3	−0.166	0.114	0.144	−0.105	0.301	0.477	0.528	0.305	0.335	0.189	0.076	0.379
Correlated change												
T2	−0.148	0.205	0.470	−0.193	−0.393	0.191	0.039	−0.323	−0.221	0.296	0.457	−0.117
T3	0.076	0.102	0.455	0.061	−0.090	0.117	0.443	−0.077	−0.077	0.151	0.608	−0.048

Problems: denotes the internalizing and externalizing problems, as specified in the columns; Cohesion: family cohesion

**Table 6:** Parameter estimates for the bivariate fixed RICLPMs modelling family communication with depressive symptoms, anxiety, and anger

Family Communication	Depressive symptoms				Anxiety				Anger			
	B	SE	*p*	*β*	B	SE	*p*	*β*	B	SE	*p*	*β*
Correlations												
Between-Person	−0.647	0.182	0.000	−0.231	−0.621	0.230	0.007	−0.256	−0.941	0.319	0.003	−0.313
T1	−0.161	0.136	0.239	−0.095	0.021	0.142	0.883	0.013	−0.253	0.232	0.276	−0.102
Cross-lagged effects												
Problems 1 → Comm. 2	0.037	0.953	0.969	0.005	0.804	1.424	0.572	0.111	0.672	0.931	0.470	0.138
Problems 2 → Comm. 3	0.037	0.953	0.969	0.003	0.804	1.424	0.572	0.064	0.672	0.931	0.470	0.114
Comm. 1 → Problems 2	−0.002	0.012	0.828	−0.036	−0.003	0.014	0.852	−0.034	0.012	0.021	0.580	0.070
Comm. 2 → Problems 3	−0.002	0.012	0.828	−0.018	−0.003	0.014	0.852	−0.022	0.012	0.021	0.580	0.063
Stability paths												
Comm. 1 → Comm. 2	−0.040	0.157	0.799	−0.050	−0.041	0.165	0.805	−0.050	−0.041	0.155	0.791	−0.051
Comm. 2 → Comm. 3	−0.040	0.157	0.799	−0.032	−0.041	0.165	0.805	−0.033	−0.041	0.155	0.791	−0.033
Problems 1 → Problems 2	−0.167	0.112	0.135	−0.271	0.040	0.202	0.841	0.057	0.309	0.170	0.069	0.303
Problems 2 → Problems 3	−0.167	0.112	0.135	−0.105	0.040	0.202	0.841	0.033	0.309	0.170	0.069	0.345
Correlated change												
T2	0.065	0.256	0.798	0.082	−0.013	0.312	0.968	−0.013	0.076	0.426	0.859	0.039
T3	−0.118	0.153	0.442	−0.072	−0.089	0.122	0.465	−0.061	−0.034	0.208	0.868	−0.016

Problems: denotes the internalizing and externalizing problems, as specified in the columns; Comm.: Family communication

As seen in Tables 4–6, in most models including family flexibility and family communication the between-person associations with internalizing and externalizing problems were negative and significant. Adolescents who reported better family functioning (indicated by higher flexibility, and family communication) compared to other adolescents, tended to also report lower internalizing and externalizing problems compared to their peers. An exception to this was the between-person associations among family cohesion and adolescent internalizing and externalizing problems; these associations were not significant.

With regard to estimates for within-family correlated change, as well as the estimates for within-family cross-lagged effects, Tables 4–6 show that there were no significant estimates. That is, whether an adolescent experienced changes in the family functioning, compared to this adolescent’s own typical family functioning, was unrelated to this adolescent’s change (either increase or decrease) in her/his internalizing and/or externalizing problems. In short, these results indicate that despite the meaningful variances on the within-family level in each variable, and in contrast to the hypotheses offered by family systems theory, the associations among family functioning and internalizing and externalizing problems are more a matter of between-family differences, than of correlated within-family fluctuations.

### Alternative Models

As planned (see link to OSF), further analyses were conducted to investigate whether models specified differently than the initial RICLPMs could provide a better fit to the data. These models were: 1) standard Cross-Lagged Panel Models (standard CLPMs), and 2) RICLPMs where the lagged coefficients were left free to vary across time intervals (instead of being fixed equal across time intervals). Fit was judged based on the same criteria used for the initial models (RMSEA, CFI, TLI). In Table 3, the alternative models that had a better fit compared to the initial models are noted with an asterisk.

The parameter estimates for those alternative models with better fit compared to the initial models are given in Tables S1-S4 in the Supplementary Material. In all models that included Depressive symptoms, as well as those including Family Cohesion (5 models in total) the more parsimonious standard CLPM provided better fit than the initial RICLPMs. As shown in the Supplementary Material, in all five standard CLPMs, significant negative cross-lagged effects emerged from Depressive symptoms at Wave 1 and at Wave 2, to family functioning at Wave 2 and at Wave 3, respectively, as well as from Anxiety and Anger to Family Cohesion. Although CLPM confounds within-, and between-family variances (Berry and Willoughby 2017; Keijsers 2016), the findings from the alternative models, in combination with the findings from the initial RI-CLPMs that only showed between-family associations, indicate that those adolescents who reported higher Depressive symptoms at Wave 1 and at Wave 2, compared to their peers, tended to also have lower family functioning at Wave 2, and at Wave 3, respectively.

Finally, regarding the unconstrained RICLPMs, five models had significantly better fit compared to the initial RICLPMs. As seen in Tables S1–S4 in the Supplementary Material, no significant within-family effects were found in any of those alternative models.

### Sensitivity Analyses

Sensitivity analyses were conducted to explore whether results are robust when controlling for adolescent sex and socioeconomic status. Please note that these analyses were not part of the initial plan, and they are not preregistered. In this study, socioeconomic status was a composite score comprising of mother education, father education, mother employment, father employment, family status, own house (vs. rent), and home density. Those variables were measured in all three waves, resulting in three measures of ses, which were then combined in one general ses variable. Table S5 in the Supplementary Material presents the fit indices for the initial models (RICLPM) and the best fitting alternative models (either RICLPM-unconstrained, or standard CLPM), after controlling for adolescent sex and socioeconomic status, by regressing the observed scores of family functioning and internalizing/externalizing problems on sex and ses. Tables S6–S8 present the results of the RICLPM models controlling for adolescent sex and family socioeconomic status. Tables S9–S12 present the parameter estimates for the best-fitting alternative models, controlling for adolescent sex and family socioeconomic status.

As can be seen by comparing those estimates with the models without covariates, only few minor changes emerged, and they mostly referred to the autoregressive stability of anger, which turned significant in the models with covariates (Tables S6–S8). One additional change was that the between-family correlation among Depressive symptoms and Family Flexibility in the alternative unconstrained RICLPM (Table S9) turned non-significant. All other parameter estimates remained largely unchanged, which indicates that the substantive results of this study hold also when controlling for adolescent sex and family socioeconomic status.

## Discussion

Adolescence is a formative period with many changes happening on the cognitive, emotional, and social spheres. Family relationships also change during adolescence. Additionally, for some adolescents adolescence is when internalizing and externalizing problems develop. Theoretically, links between family functioning, and internalizing and externalizing problems are expected to take place on the within-family level. Yet, most developmental research thus far has failed to adequately focus on within-family processes. This study examined some of the key premises of family systems theory, that within-family changes in the family system would be bidirectionally linked to within-child changes in internalizing and externalizing problems. Following an ongoing methodological discussion on the interpretation of the CLPM (Berry and Willoughby 2017; Hamaker et al. 2015; Keijsers 2016), these longitudinal dynamic associations were tested at the within-family level, while controlling for stable differences and associations at the between-family level (e.g., Keijsers 2016). Even though the expected significant negative associations among all variables on the between-family level were found, no significant associations emerged at the within-family level. Thus, even though adolescents who grow up in families with worse communication, lower cohesion, and lower flexibility seem to be at higher risk for adaptation problems, within-family changes in family functioning were unrelated to child adaptation, which is in contrast to the pre-registered hypotheses. Applying standard CLPMs as an alternative and more parsimonious approach revealed significant negative cross-lagged effects of internalizing and externalizing problems on family functioning, but not vice versa. These results suggest that how families become more different from each other over time can be predicted from preceding differences in child adaptation. However, the interpretation of such statistical effects is an ongoing discussion by itself [for example, Berry and Willoughby (2017) label such effects an uninterpretable blend].

### Dynamic Associations Among Family Functioning and Internalizing and Externalizing Problems: Not a Within-Person Story

Based on previous theorizing and extant research, the hypotheses of this study were that family functioning would have a significant, longitudinal, negative within-family effect on internalizing (Queen et al. 2013) and externalizing problems (Elgar et al. 2013). Also, it was predicted that internalizing and externalizing problems would have a significant, longitudinal, negative within-family effect on family functioning. However, no significant effects in support of any of these predictions were found, despite the relatively large sample size (*N* = 480).

The absence of significant within-family effects (both cross-lagged, and correlated change) indicates that the associations among family functioning and internalizing and externalizing problems are not a within-family process in which the child and family system affect each other. In contrast, the presence of significant cross-lagged effects in the CLPMs, might indicate that the dynamic associations among family functioning and adolescent internalizing and externalizing problems might be a matter of stable differences between families in the emotional climate, which can be predicted by child adaptation the preceding months. It appears that what matters is the relative standing of an adolescent’s family functioning in relation to other peers when it comes to understanding who will develop internalizing and externalizing problems, and vice versa. In this sense, the current results are in accordance with the results of previous studies, which used between-family designs (Branje et al. 2010). Adolescents who experience more internalizing and externalizing problems relative to their peers are also expected to experience worse family functioning the following months. This study shows that it is possible to identify who, among adolescents, might be more in need of a psychological and/or family therapy intervention, given the longitudinal effects found among symptoms of depression and family functioning.

From a theoretical point of view, the absence of significant within-family effects may help clarify the theoretical expectations on how family functioning and internalizing/externalizing problems develop in middle to late adolescence, and how it affects the psychological well-being of individual adolescents. Given that the current study applied an explicit disaggregation of between-, and within-family sources of variance in investigating order of effects, it stands to point that most family processes during adolescence become meaningful only when the rest of the group is taken into account. Thus, families differ from each other, which may provide insights into who is at risk. The Circumplex Model (Olson 2011), on which the current study was based, may work well as a static description of differences between families, yet, no evidence for the hypothesized processes within-families was found. Newer models may be needed that make explicit developmental hypotheses regarding how the family processes, and fluctuations therein, affect the psychological well-being of individual members in the family. What is urgently needed in this respect is a better clarification also of the time scale at which these dynamic processes take place. For instance, in dynamic system approaches (e.g., Granic and Patterson 2006), the accumulation of short-term micro-dynamics should yield longer term divergent change in families and child adaptation. But the short- and longer-term patterns may be non-linearly related. The mere fact that reciprocal effects were not found in the present study may also mean that the focal time frame is discordant with the time scale at which these mechanisms take place (Keijsers and van Roekel 2018). Given that there is now a much better access to the statistical techniques to explore such hypotheses (e.g., Dynamic Structural Equation Modeling, Asparouhov et al. 2018; Continuous Time Structural Equation Modeling, Voelkle et al. 2012), and novel methods to collect data into short-term dynamics, such as Experience Sampling (Keijsers and van Roekel 2018), future studies and conceptual developments may help move the field of family systems forward.

### Alternative Dynamic Associations Among Family Functioning and Internalizing/Externalizing Problems

Applying standard CLPMs as an alternative approach to investigate the order of effects among family functioning and internalizing/externalizing problems in adolescence, consistent, significant, and negative cross-lagged effects were found, from internalizing/externalizing problems to family functioning over intervals of six months. Yet, although statistically it is meaningful to see that adolescent adaptation problems may predict interfamily differences over time in family functioning, the theoretical interpretation of CLPM cross-lagged effects (e.g., are they between-family?) is a question of methodological debate (Berry and Willoughby 2017).

Statistically, adolescents that experienced more symptoms of depression compared to their peers, were more likely to experience a deterioration in their family flexibility, family cohesion, and family communication six months later, while controlling for different types of stability. Even though the exact meaning of CLPM is now an issue of debate (Berry and Willoughby 2017; Keijsers 2016), the current findings do help to clarify extant cross-sectional research on the associations among family functioning and adolescent symptoms of depression (Elgar et al. 2013), by elucidating the direction of statistical prediction. Importantly, the opposite direction of effects, that is, effects of family functioning on symptoms of depression, was not found. This is in accordance with recent longitudinal research that has showed that during adolescence “child effects” become stronger compared to “parent effects” (e.g., Georgiou and Fanti 2014; for a review, see Meeus 2016), but it is in disagreement with other studies showing bidirectional effects among adolescent symptoms of depression and parent-adolescent relationship quality (Branje et al. 2010). Stated otherwise, in line with the view that parents and family lose part of their significance compared to other aspects of the adolescent’s psychosocial spheres (e.g., the role of peers, other social networks), the current pre-registered study offered no evidence for effects from family functioning to adolescent symptoms of depression. Therefore, the present results show that family as a system might not play a significant causal role in developing depressive symptoms during adolescence.

Furthermore, the results showed that adolescents who experienced more anxiety and more anger, compared to their peers, were the most probable to experience a decrease in their family cohesion. Similar findings have been obtained in extant research (e.g., Jozefiak and Wallander 2016). Similar to the findings regarding symptoms of depression, these findings are in agreement with the “child effects” view that adolescents play an ever stronger role in the development of their environment, as they grow older (e.g., Meeus 2016). In contrast, changes in the relative standing of families in family cohesion do not appear to have an effect of adolescent anger and symptoms of anxiety.

### Limitations, Strengths, and Future Directions

Several limitations must be taken into account when considering the results of this study. First, even though self-report can be an adequate method in obtaining insights regarding internalizing problems, family functioning as well as externalizing problems could also be measured with other-reported data and/or observations, to obtain a more objective view. Second, the causal processes between family functioning and adolescent adaptation may take place at longer or shorter time intervals, making it impossible to detect them with the fixed time lags used in this study. An alternative approach could be to have time lags with multiple lengths in one study design (Keijsers and van Roekel 2018) and apply continuous time models (Voelkle et al. 2012) to assess how the effect depends on the length of the time interval, and when the effect is the strongest. Moreover, it could be that the duration of the study was relatively small, making it impossible to detect the longer term (developmental changes in) within-family processes. Following the same participants throughout adolescence, preferably with measurement burst with shorter intervals, might provide better insights in the dynamic interplay among family functioning and internalizing and externalizing problems at the macro-time scale. For instance, to tap into influences between family functioning and adolescent adaptation measurements at a meso-, or a micro-time scale, daily diaries or Experience Sampling, may be useful additional methodologies.

Notwithstanding these limitations, this study has several strengths and it offers important insights in the dynamic interplay among family functioning and internalizing and externalizing problems during adolescence. First, the application of newly developed statistical techniques on longitudinal data, which allows to differentiate within-family from between-family effects, has specific strengths in family research, as such research is often plagued by challenges of differentiating gene-environment effects, environmental effects, or other stable confounders, from the actual influences family functioning has on the child. By controlling for all stable differences in family functioning and in the adolescent outcomes, by default, all stable confounders are controlled for, whether they are included in the study design or not. This allows to examine relevant theories on the ecological level these theories are postulated, that is, the within-family level, and provides a much more stringent test for the influences of families on children. Earlier research has illustrated that this may also lead to paradoxical findings, for instance, positive associations at the between-family level, but negative associations at the within-family level (e.g., Dietvorst et al. 2018). Thus, a new wave of research, with novel analytical methods, may help to critically evaluate and refine existing ideas. Second, the theoretical background, the rationale, and the plan of analyses of this study were all pre-registered, based on state-of-the-art methodological writings, and followed in detail in this study. This is important because in this way all analyses are well-thought in advance, and they are tailored to test pre-specified hypotheses, instead of exploring associations in a big dataset. Applying open science practices (Nosek et al. 2015) is a promising route to improving psychological science, and this study sets an example of how the analytical plan for RICLPM can also be preregistered. Third, a relatively large sample of a broadly understudied population was used. Even though null results are difficult to test (Ferguson and Heene 2012), the large sample indicates that the null results found in this study are legitimate.

Overall, this study has been designed to provide a more stringent test of existing theoretical hypotheses regarding the link of family functioning with child internalizing and externalizing problems. It is one example of a larger movement in which it is advocated that future research should be designed to match the theoretical time scales to the analytical designs (Keijsers and van Roekel 2018). Theory ultimately should determine what the adequate time scale of observation is, and in this respect, building a solid theoretical basis, which is explicit about the ecological level and time scale of developmental processes, is a major challenge for future research.

## Conclusion

Adolescence can be a challenging period for some adolescents, because of the increased risk for the development of internalizing and/or externalizing problems. Even though parent-adolescent relationships change during adolescence (Branje et al. 2012), as shown, for example, by the increased parent-adolescent conflict intensity (Mastrotheodoros et al. 2019a), family remains an influential context for adolescent development. Family systems theory posits that family functioning is important for adolescent adaptation, a proposition that is located on the within-family level. Thus, if family functioning changes within one family, for instance, the cohesion decreases, this should affect the adolescent within that same family. To test this proposition appropriately, it is necessary to explicitly disaggregate variances due to between-family differences, from the fluctuations and changes, as well as their associations, at the within-family level (e.g., Hamaker et al. 2015; Keijsers 2016).

This study investigated the longitudinal dynamic associations between family functioning and internalizing and externalizing problems, on the within-family level, while controlling for stable between-family differences and associations. The null results on the within-family level indicate that, during adolescence, fluctuations in family functioning as well as in internalizing/externalizing problems are not associated with each other at the 6-month time interval. At the same time, significant effects in the CLPM models suggest that statistical prediction is still possible—mainly from adolescent adaptation to family functioning. For example, based on the results of the current study, it is suggested that adolescents who develop more symptoms of depression are statistically in higher risk for experiencing decreases in family functioning the following months compared to their peers who do not experience increases in depressive symptoms. However, this statistical link is most likely not a causal one.

To conclude, the results of this study have important theoretical and practical implications. The null results on the within-person level call for further empirical research and perhaps even theoretical refinement regarding the ecological level and time scale at which developmental processes, such as the ones studied here, take place (e.g., Keijsers and van Roekel 2018). It appears that, even though within-family fluctuations in family functioning are largely inconsequential for adolescent internalizing and externalizing problems, robust between-family associations exist in how adolescent adaptation and family functioning relate to each other. In families with more flexibility, cohesion, and communication, adolescents are on average better adapted. But, to date, we cannot conclude that this link is due to within-family dynamic processes between changes in family functioning and adolescent adaptation. Until a better understanding of within-family processes is reached, focusing on those adolescents with higher than average symptoms of depression, anxiety, and anger may help to identify those among youth that are most in need for family interventions.

## Supplementary information

Supplementary Materials
