# Supplementary material for: Family Functioning and Adolescent Internalizing and Externalizing Problems: Disentangling between-, and Within-Family Associations
Source: J Youth Adolesc. 2019 Aug 5;49(4):804–17. doi: 10.1007/s10964-019-01094-z (PMC7105424; doi:10.1007/s10964-019-01094-z)
Supplement: Supplementary file 1 — Supplementary Materials [file 10964_2019_1094_MOESM1_ESM.docx]

Table S1

Parameter Estimates from the Alternative Models for Family Flexibility that Fit Better Compared to the Fixed RICLPMs.

| Family Flexibility | Depressive symptoms – CLPM | | | |  | Depressive symptoms– RICLPM-free | | | |  | Anxiety – RICLPM-free | | | | |
| --- | --- | --- | --- | --- | --- | --- | --- | --- | --- | --- | --- | --- | --- | --- | --- |
|  | B | SE | *p* | *β* |  | B | SE | *p* | *β* |  | B | SE | *p* | *β* |  |
| Correlations |  |  |  |  |  |  |  |  |  |  |  |  |  |  |  |
| Between-Person | - | - | - | - |  | -0.310 | 0.144 | 0.032 | -0.158 |  | -0.269 | 0.165 | 0.103 | -0.166 |  |
| T1 | -0.182 | 0.128 | 0.155 | -0.062 |  | 0.083 | 0.122 | 0.498 | 0.085 |  | -0.008 | 0.127 | 0.947 | -0.008 |  |
| Cross-Lagged Effects |  |  |  |  |  |  |  |  |  |  |  |  |  |  |  |
| Problems 1 🡪 Flexibility 2 | -0.726 | 0.213 | 0.001 | -0.110 |  | 0.014 | 1.023 | 0.989 | 0.002 |  | 0.748 | 0.958 | 0.435 | 0.117 |  |
| Problems 2 🡪 Flexibility 3 | -0.726 | 0.213 | 0.001 | -0.103 |  | -0.350 | 1.153 | 0.762 | -0.041 |  | -1.180 | 1.114 | 0.289 | -0.160 |  |
| Flexibility 1 🡪 Problems 2 | 0.001 | 0.005 | 0.849 | 0.006 |  | 0.045 | 0.028 | 0.100 | 0.346 |  | 0.008 | 0.025 | 0.751 | 0.052 |  |
| Flexibility 2 🡪 Problems 3 | 0.001 | 0.005 | 0.849 | 0.006 |  | -0.019 | 0.016 | 0.226 | -0.111 |  | -0.013 | 0.014 | 0.340 | -0.080 |  |
| Stability Paths |  |  |  |  |  |  |  |  |  |  |  |  |  |  |  |
| Flexibility 1 🡪 Flexibility 2 | 0.434 | 0.043 | 0.000 | 0.424 |  | -0.229 | 0.175 | 0.191 | -0.218 |  | -0.239 | 0.165 | 0.147 | -0.228 |  |
| Flexibility 2 🡪 Flexibility 3 | 0.434 | 0.043 | 0.000 | 0.427 |  | -0.072 | 0.135 | 0.593 | -0.068 |  | -0.096 | 0.139 | 0.489 | -0.090 |  |
| Problems 1 🡪 Problems 2 | 0.610 | 0.042 | 0.000 | 0.639 |  | -0.267 | 0.221 | 0.227 | -0.308 |  | 0.116 | 0.169 | 0.495 | 0.125 |  |
| Problems 2 🡪 Problems 3 | 0.610 | 0.042 | 0.000 | 0.548 |  | 0.300 | 0.157 | 0.057 | 0.216 |  | 0.453 | 0.134 | 0.001 | 0.393 |  |
| Correlated Change |  |  |  |  |  |  |  |  |  |  |  |  |  |  |  |
| T2 | -0.002 | 0.068 | 0.981 | -0.001 |  | 0.122 | 0.204 | 0.551 | 0.157 |  | -0.057 | 0.186 | 0.760 | -0.058 |  |
| T3 | -0.002 | 0.068 | 0.981 | -0.001 |  | -0.053 | 0.102 | 0.605 | -0.042 |  | -0.106 | 0.098 | 0.277 | -0.095 |  |

Note: CLPM: Cross-Lagged Panel Model; RICLPM-free: Random-Intercept Cross-Lagged Panel Model with no time-invariance constrains; Problems: Internalizing/Externalizing Problems, as specified in the columns; Flexibility: Family Flexibility.

Table S2

Parameter Estimates from the Alternative Models for Family Cohesion that Fit Better Compared to the Fixed RICLPMs.

| Family Cohesion | Depressive symptoms – CLPM | | | |  | Anxiety – CLPM | | | |  | Anxiety – RICLPM-free | | | |  | Anger – CLPM | | | |
| --- | --- | --- | --- | --- | --- | --- | --- | --- | --- | --- | --- | --- | --- | --- | --- | --- | --- | --- | --- |
|  | B | SE | p | β |  | B | SE | p | β |  | B | SE | p | β |  | B | SE | p | β |
| Correlations |  |  |  |  |  |  |  |  |  |  |  |  |  |  |  |  |  |  |  |
| Between-Person | - | - | - | - |  | - | - | - | - |  | -0.302 | 0.190 | 0.112 | -0.184 |  | - | - | - | - |
| T1 | -0.274 | 0.161 | 0.089 | -0.085 |  | -0.336 | 0.145 | 0.021 | -0.115 |  | -0.055 | 0.155 | 0.723 | -0.042 |  | -0.534 | 0.239 | 0.025 | -0.128 |
| Cross-Lagged Effects |  |  |  |  |  |  |  |  |  |  |  |  |  |  |  |  |  |  |  |
| Problems 1 🡪 Cohesion 2 | -0.582 | 0.214 | 0.006 | -0.087 |  | -0.513 | 0.251 | 0.041 | -0.070 |  | 0.160 | 1.011 | 0.874 | 0.023 |  | -0.549 | 0.160 | 0.001 | -0.107 |
| Problems 2 🡪 Cohesion 3 | -0.582 | 0.214 | 0.006 | -0.079 |  | -0.513 | 0.251 | 0.041 | -0.065 |  | -0.888 | 1.118 | 0.427 | -0.115 |  | -0.549 | 0.160 | 0.001 | -0.101 |
| Cohesion 1 🡪 Problems 2 | 0.002 | 0.005 | 0.655 | 0.016 |  | -0.002 | 0.005 | 0.659 | -0.016 |  | -0.001 | 0.018 | 0.961 | -0.007 |  | 0.003 | 0.007 | 0.681 | 0.016 |
| Cohesion 2 🡪 Problems 3 | 0.002 | 0.005 | 0.655 | 0.013 |  | -0.002 | 0.005 | 0.659 | -0.015 |  | -0.012 | 0.017 | 0.468 | -0.079 |  | 0.003 | 0.007 | 0.681 | 0.015 |
| Stability Paths |  |  |  |  |  |  |  |  |  |  |  |  |  |  |  |  |  |  |  |
| Cohesion 1 🡪 Cohesion 2 | 0.553 | 0.040 | 0.000 | 0.584 |  | 0.553 | 0.040 | 0.000 | 0.581 |  | 0.079 | 0.142 | 0.578 | 0.084 |  | 0.551 | 0.039 | 0.000 | 0.577 |
| Cohesion 2 🡪 Cohesion 3 | 0.553 | 0.040 | 0.000 | 0.526 |  | 0.553 | 0.040 | 0.000 | 0.528 |  | 0.105 | 0.147 | 0.475 | 0.103 |  | 0.551 | 0.039 | 0.000 | 0.527 |
| Problems 1 🡪 Problems 2 | 0.605 | 0.035 | 0.000 | 0.633 |  | 0.620 | 0.039 | 0.000 | 0.636 |  | 0.118 | 0.178 | 0.508 | 0.128 |  | 0.575 | 0.040 | 0.000 | 0.581 |
| Problems 2 🡪 Problems 3 | 0.605 | 0.035 | 0.000 | 0.546 |  | 0.620 | 0.039 | 0.000 | 0.592 |  | 0.447 | 0.154 | 0.004 | 0.388 |  | 0.575 | 0.040 | 0.000 | 0.580 |
| Correlated Change |  |  |  |  |  |  |  |  |  |  |  |  |  |  |  |  |  |  |  |
| T2 | -0.056 | 0.074 | 0.455 | -0.031 |  | -0.120 | 0.066 | 0.068 | -0.071 |  | -0.199 | 0.171 | 0.243 | -0.180 |  | -0.067 | 0.089 | 0.453 | -0.026 |
| T3 | -0.056 | 0.074 | 0.455 | -0.030 |  | -0.120 | 0.066 | 0.068 | -0.075 |  | -0.073 | 0.102 | 0.474 | -0.061 |  | -0.067 | 0.089 | 0.453 | -0.030 |

Note: CLPM: Cross-Lagged Panel Model; RICLPM-free: Random-Intercept Cross-Lagged Panel Model with no time-invariance constrains; Problems: Internalizing/Externalizing Problems, as specified in the columns; Cohesion: Family Cohesion.

Table S3

Parameter Estimates from the Alternative Models for Family Communication that Fit Better Compared to the Fixed RICLPMs.

| Family Communication | Depressive symptoms – CLPM | | | |  | Anxiety – CLPM | | | |  | Anxiety – RICLPM-free | | | |  | Anger – CLPM | | | |
| --- | --- | --- | --- | --- | --- | --- | --- | --- | --- | --- | --- | --- | --- | --- | --- | --- | --- | --- | --- |
|  | B | SE | *p* | *β* |  | B | SE | *p* | *β* |  | B | SE | *p* | *β* |  | B | SE | *p* | *β* |
| Correlations |  |  |  |  |  |  |  |  |  |  |  |  |  |  |  |  |  |  |  |
| Between-Person | - | - | - | - |  | - | - | - | - |  | -0.609 | 0.221 | 0.006 | -0.277 |  | - | - | - | - |
| T1 | -0,700 | 0,225 | 0,002 | -0,170 |  | -0.487 | 0.206 | 0.018 | -0.130 |  | 0.002 | 0.202 | 0.993 | 0.001 |  | -0.950 | 0.279 | 0.001 | -0.179 |
| Cross-Lagged Effects |  |  |  |  |  |  |  |  |  |  |  |  |  |  |  |  |  |  |  |
| Problems 1 🡪 Comm. 2 | -0,764 | 0,267 | 0,004 | -0,088 |  | -0.281 | 0.304 | 0.356 | -0.030 |  | 1.647 | 1.394 | 0.238 | 0.209 |  | -0.264 | 0.242 | 0.275 | -0.040 |
| Problems 2 🡪 Comm. 3 | -0,764 | 0,267 | 0,004 | -0,077 |  | -0.281 | 0.304 | 0.356 | -0.026 |  | -0.388 | 1.422 | 0.785 | -0.037 |  | -0.264 | 0.242 | 0.275 | -0.036 |
| Comm. 1 🡪 Problems 2 | -0,003 | 0,004 | 0,440 | -0,028 |  | -0.007 | 0.004 | 0.070 | -0.068 |  | -0.018 | 0.016 | 0.280 | -0.169 |  | -0.003 | 0.005 | 0.506 | -0.024 |
| Comm. 2 🡪 Problems 3 | -0,003 | 0,004 | 0,440 | -0,024 |  | -0.007 | 0.004 | 0.070 | -0.063 |  | 0.011 | 0.013 | 0.363 | 0.085 |  | -0.003 | 0.005 | 0.506 | -0.023 |
| Stability Paths |  |  |  |  |  |  |  |  |  |  |  |  |  |  |  |  |  |  |  |
| Comm. 1 🡪 Comm. 2 | 0,573 | 0,041 | 0,000 | 0,596 |  | 0.583 | 0.040 | 0.000 | 0.607 |  | -0.089 | 0.154 | 0.565 | -0.099 |  | 0.581 | 0.040 | 0.000 | 0.601 |
| Comm. 2 🡪 Comm. 3 | 0,573 | 0,041 | 0,000 | 0,523 |  | 0.583 | 0.040 | 0.000 | 0.531 |  | 0.160 | 0.201 | 0.426 | 0.129 |  | 0.581 | 0.040 | 0.000 | 0.531 |
| Problems 1 🡪 Problems 2 | 0,600 | 0,036 | 0,000 | 0,626 |  | 0.613 | 0.038 | 0.000 | 0.630 |  | 0.131 | 0.165 | 0.426 | 0.143 |  | 0.576 | 0.040 | 0.000 | 0.581 |
| Problems 2 🡪 Problems 3 | 0,600 | 0,036 | 0,000 | 0,544 |  | 0.613 | 0.038 | 0.000 | 0.586 |  | 0.479 | 0.125 | 0.000 | 0.414 |  | 0.576 | 0.040 | 0.000 | 0.583 |
| Correlated Change |  |  |  |  |  |  |  |  |  |  |  |  |  |  |  |  |  |  |  |
| T2 | -0,078 | 0,090 | 0,383 | -0,034 |  | -0.136 | 0.083 | 0.101 | -0.064 |  | -0.071 | 0.227 | 0.756 | -0.058 |  | -0.235 | 0.129 | 0.068 | -0.072 |
| T3 | -0,078 | 0,090 | 0,383 | -0,032 |  | -0.136 | 0.083 | 0.101 | -0.064 |  | -0.103 | 0.121 | 0.394 | -0.063 |  | -0.235 | 0.129 | 0.068 | -0.079 |

Note: CLPM: Cross-Lagged Panel Model; RICLPM-free: Random-Intercept Cross-Lagged Panel Model with no time-invariance constrains; Problems: Internalizing/Externalizing Problems, as specified in the columns; Comm.: Family Communication.

Table S4

Parameter Estimates from the Alternative Model for Family Communication and Anger, which Fit Better Compared to the Fixed RICLPMs.

| Family Communication | Anger – RICLPM-free | | | |
| --- | --- | --- | --- | --- |
|  | B | SE | *p* | *β* |
| Correlations |  |  |  |  |
| Between-Person | -0.736 | 0.235 | 0.002 | -0.243 |
| T1 | -0.369 | 0.226 | 0.102 | -0.174 |
| Cross-Lagged Effects |  |  |  |  |
| Problems 1 🡪 Comm. 2 | 0.510 | 0.856 | 0.551 | 0.086 |
| Problems 2 🡪 Comm. 3 | 0.137 | 0.776 | 0.860 | 0.019 |
| Comm. 1 🡪 Problems 2 | -0.012 | 0.022 | 0.594 | -0.071 |
| Comm. 2 🡪 Problems 3 | 0.000 | 0.015 | 0.995 | 0.001 |
| Stability Paths |  |  |  |  |
| Comm. 1 🡪 Comm. 2 | -0.133 | 0.154 | 0.387 | -0.140 |
| Comm. 2 🡪 Comm. 3 | 0.150 | 0.164 | 0.361 | 0.118 |
| Problems 1 🡪 Problems 2 | 0.060 | 0.143 | 0.673 | 0.058 |
| Problems 2 🡪 Problems 3 | 0.296 | 0.099 | 0.003 | 0.309 |
| Correlated Change |  |  |  |  |
| T2 | -0.240 | 0.287 | 0.405 | -0.117 |
| T3 | -0.220 | 0.184 | 0.233 | -0.092 |

Note: RICLPM-free: Random-Intercept Cross-Lagged Panel Model with no time-invariance constrains; Problems: Internalizing/Externalizing Problems, as specified in the columns; Comm.: Family Communication.

Appendix

Sensitivity Analyses

*Table S5*

Model Fit Indices for Initial Models and Best Fitting Alternative Models, Controlling for Adolescent sex and family socioeconomic status.

|  | Model Type | *χ*^2^ | *df* | CFI | TLI | RMSEA |
| --- | --- | --- | --- | --- | --- | --- |
| Flexibility - Depressive Symptoms | RICLPM-fixed | 13.814 | 5 | 0.989 | 0.942 | 0.061 |
| Flexibility - Depressive Symptoms* | CLPM | 16.868 | 7 | 0.988 | 0.954 | 0.054 |
| Flexibility - Depressive Symptoms* | RICLPM-free | 2.789 | 1 | 0.998 | 0.941 | 0.061 |
|  |  |  |  |  |  |  |
| Flexibility - Anxiety | RICLPM-fixed | 19.024 | 5 | 0.983 | 0.907 | 0.076 |
| Flexibility - Anxiety* | RICLPM-free | 1.816 | 1 | 0.999 | 0.973 | 0.041 |
|  |  |  |  |  |  |  |
| Flexibility - Anger | RICLPM-fixed | 11.131 | 5 | 0.992 | 0.956 | 0.051 |
|  |  |  |  |  |  |  |
| Cohesion - Depressive Symptoms | RICLPM-fixed | 18.662 | 5 | 0.984 | 0.912 | 0.075 |
| Cohesion - Depressive Symptoms* | CLPM | 15.215 | 7 | 0.990 | 0.962 | 0.049 |
|  |  |  |  |  |  |  |
| Cohesion - Anxiety | RICLPM-fixed | 17.854 | 5 | 0.984 | 0.916 | 0.073 |
| Cohesion - Anxiety* | CLPM | 15.006 | 7 | 0.990 | 0.963 | 0.049 |
| Cohesion - Anxiety* | RICLPM-free | 5.271 | 1 | 0.995 | 0.861 | 0.094 |
|  |  |  |  |  |  |  |
| Cohesion - Anger | RICLPM-fixed | 17.539 | 5 | 0.984 | 0.912 | 0.072 |
| Cohesion - Anger* | CLPM | 11.657 | 7 | 0.994 | 0.977 | 0.037 |
|  |  |  |  |  |  |  |
| Communication - Depressive Symptoms | RICLPM-fixed | 14.561 | 5 | 0.989 | 0.942 | 0.063 |
| Communication - Depressive Symptoms* | CLPM | 5.251 | 7 | 1.000 | 1.008 | 0.000 |
|  |  |  |  |  |  |  |
| Communication - Anxiety | RICLPM-fixed | 20.710 | 5 | 0.982 | 0.903 | 0.081 |
| Communication – Anxiety* | CLPM | 14.507 | 7 | 0.991 | 0.967 | 0.047 |
| Communication - Anxiety* | RICLPM-free | 0.620 | 1 | 1.000 | 1.012 | 0.000 |
|  |  |  |  |  |  |  |
| Communication - Anger | RICLPM-fixed | 13.514 | 5 | 0.990 | 0.943 | 0.060 |
| Communication - Anger* | CLPM | 12.374 | 7 | 0.993 | 0.975 | 0.040 |
| Communication - Anger* | RICLPM-free | 4.712 | 1 | 0.995 | 0.877 | 0.088 |
|  |  |  |  |  |  |  |

*Note:* RICLPM-fixed: Random-Intercept Cross-Lagged Panel Models with time invariance constrains on the autoregressive stabilities and the cross-lagged effects. CLPM: Cross Lagged Panel Model; RICLPM-free: fully unconstrained Random Intercept Cross Lagged Pan el Model; CFI: Comparative Fit Index; TLI: Tucker-Lewis Index; RMSEA: Root Mean Square Error of Approximation.

* This model has better fit than the initial/original model, and therefore alternative model results are presented in the Supplementary Material.

*Table S6*

Parameter Estimates for the bivariate fixed RICLPMs modelling Family Flexibility with Depressive symptoms, Anxiety, and Anger, controlling for adolescent sex and family socioeconomic status.

| Family Flexibility | | | Depressive symptoms | | |  | Anxiety | | | |  | Anger | | | | | |
| --- | --- | --- | --- | --- | --- | --- | --- | --- | --- | --- | --- | --- | --- | --- | --- | --- | --- |
|  | B | SE | | *p* | *β* |  | B | SE | *p* | *β* | | |  | B | SE | *p* | *β* |
| Correlations |  |  | |  |  |  |  |  |  |  | | |  |  |  |  |  |
| Between-Person | -0.228 | 0.117 | | 0.050 | -0.121 |  | -0.266 | 0.134 | 0.048 | -0.161 | | |  | -0.248 | 0.193 | 0.199 | -0.120 |
| T1 | -0.118 | 0.081 | | 0.145 | -0.101 |  | -0.151 | 0.088 | 0.085 | -0.133 | | |  | -0.292 | 0.161 | 0.070 | -0.171 |
| Cross-Lagged Effects |  |  | |  |  |  |  |  |  |  | | |  |  |  |  |  |
| Problems 1 🡪 Flex. 2 | -0.053 | 0.631 | | 0.933 | -0.009 |  | 0.398 | 0.815 | 0.625 | 0.064 | | |  | -0.386 | 0.508 | 0.447 | -0.090 |
| Problems 2 🡪 Flex. 3 | -0.053 | 0.631 | | 0.933 | -0.005 |  | 0.398 | 0.815 | 0.625 | 0.043 | | |  | -0.386 | 0.508 | 0.447 | -0.094 |
| Flex. 1 🡪 Problems 2 | 0.010 | 0.013 | | 0.446 | 0.096 |  | 0.004 | 0.013 | 0.786 | 0.033 | | |  | -0.007 | 0.020 | 0.718 | -0.030 |
| Flex. 2 🡪 Problems 3 | 0.010 | 0.013 | | 0.446 | 0.060 |  | 0.004 | 0.013 | 0.786 | 0.024 | | |  | -0.007 | 0.020 | 0.718 | -0.034 |
| Stability Paths |  |  | |  |  |  |  |  |  |  | | |  |  |  |  |  |
| Flex. 1 🡪 Flex. 2 | -0.128 | 0.132 | | 0.331 | -0.130 |  | -0.149 | 0.130 | 0.251 | -0.152 | | |  | -0.114 | 0.146 | 0.437 | -0.111 |
| Flex. 2 🡪 Flex. 3 | -0.128 | 0.132 | | 0.331 | -0.125 |  | -0.149 | 0.130 | 0.251 | -0.145 | | |  | -0.114 | 0.146 | 0.437 | -0.114 |
| Problems 1 🡪 Problems 2 | -0.157 | 0.103 | | 0.127 | -0.251 |  | 0.012 | 0.170 | 0.944 | 0.017 | | |  | 0.323 | 0.163 | 0.048 | 0.308 |
| Problems 2 🡪 Problems 3 | -0.157 | 0.103 | | 0.127 | -0.100 |  | 0.012 | 0.170 | 0.944 | 0.009 | | |  | 0.323 | 0.163 | 0.048 | 0.366 |
| Correlated Change |  |  | |  |  |  |  |  |  |  | | |  |  |  |  |  |
| T2 | 0.131 | 0.170 | | 0.440 | 0.191 |  | 0.072 | 0.181 | 0.690 | 0.094 | | |  | -0.296 | 0.290 | 0.308 | -0.171 |
| T3 | 0.034 | 0.094 | | 0.721 | 0.030 |  | -0.022 | 0.086 | 0.795 | -0.022 | | |  | -0.066 | 0.131 | 0.617 | -0.044 |

*Note*. Problems: Denotes the Internalizing and Externalizing Problems, as specified in the columns; Flex.: Family Flexibility.

*Table S7*

Parameter Estimates for the bivariate fixed RICLPMs modelling Family Cohesion with Depressive symptoms, Anxiety, and Anger, controlling for adolescent sex and family socioeconomic status.

| Family Cohesion | | Depressive symptoms | | | |  | Anxiety | | | |  | Anger | | | | | | |
| --- | --- | --- | --- | --- | --- | --- | --- | --- | --- | --- | --- | --- | --- | --- | --- | --- | --- | --- |
|  | B | | SE | *p* | *β* |  | B | SE | *p* | *β* | | |  | B | SE | *p* | *β* |  |
| Correlations |  | |  |  |  |  |  |  |  |  | | |  |  |  |  |  |  |
| Between-Person | -0.263 | | 0.162 | 0.105 | -0.141 |  | -0.081 | 0.175 | 0.643 | -0.059 | | |  | -0.241 | 0.265 | 0.363 | -0.119 |  |
| T1 | -0.110 | | 0.112 | 0.325 | -0.079 |  | -0.204 | 0.137 | 0.137 | -0.142 | | |  | -0.117 | 0.264 | 0.658 | -0.056 |  |
| Cross-Lagged Effects |  | |  |  |  |  |  |  |  |  | | |  |  |  |  |  |  |
| Problems 1 🡪 Cohesion 2 | -0.237 | | 0.785 | 0.763 | -0.033 |  | -1.250 | 0.724 | 0.084 | -0.175 | | |  | -0.625 | 0.556 | 0.262 | -0.133 |  |
| Problems 2 🡪 Cohesion 3 | -0.237 | | 0.785 | 0.763 | -0.022 |  | -1.250 | 0.724 | 0.084 | -0.177 | | |  | -0.625 | 0.556 | 0.262 | -0.141 |  |
| Cohesion 1 🡪 Problems 2 | -0.005 | | 0.014 | 0.731 | -0.059 |  | -0.021 | 0.016 | 0.195 | -0.150 | | |  | -0.011 | 0.024 | 0.648 | -0.052 |  |
| Cohesion 2 🡪 Problems 3 | -0.005 | | 0.014 | 0.731 | -0.036 |  | -0.021 | 0.016 | 0.195 | -0.152 | | |  | -0.011 | 0.024 | 0.648 | -0.056 |  |
| Stability Paths |  | |  |  |  |  |  |  |  |  | | |  |  |  |  |  |  |
| Cohesion 1 🡪 Cohesion 2 | 0.161 | | 0.126 | 0.201 | 0.171 |  | 0.166 | 0.108 | 0.124 | 0.166 | | |  | 0.142 | 0.135 | 0.292 | 0.148 |  |
| Cohesion 2 🡪 Cohesion 3 | 0.161 | | 0.126 | 0.201 | 0.166 |  | 0.166 | 0.108 | 0.124 | 0.171 | | |  | 0.142 | 0.135 | 0.292 | 0.144 |  |
| Problems 1 🡪 Problems 2 | -0.156 | | 0.112 | 0.162 | -0.246 |  | 0.339 | 0.331 | 0.306 | 0.345 | | |  | 0.349 | 0.174 | 0.045 | 0.332 |  |
| Problems 2 🡪 Problems 3 | -0.156 | | 0.112 | 0.162 | -0.102 |  | 0.339 | 0.331 | 0.306 | 0.342 | | |  | 0.349 | 0.174 | 0.045 | 0.393 |  |
| Correlated Change |  | |  |  |  |  |  |  |  |  | | |  |  |  |  |  |  |
| T2 | -0.141 | | 0.205 | 0.492 | -0.175 |  | -0.396 | 0.170 | 0.020 | -0.314 | | |  | -0.246 | 0.284 | 0.386 | -0.128 |  |
| T3 | 0.071 | | 0.101 | 0.481 | 0.057 |  | -0.104 | 0.096 | 0.276 | -0.088 | | |  | -0.088 | 0.145 | 0.541 | -0.054 |  |

*Note*. Problems: Denotes the Internalizing and Externalizing Problems, as specified in the columns; Cohesion: Family Cohesion.

*Table S8*

Parameter Estimates for the bivariate fixed RICLPMs modelling Family Communication with Depressive symptoms, Anxiety, and Anger.

| Family Communication | | | Depressive symptoms | | |  | Anxiety | | | |  | Anger | | | | | | |
| --- | --- | --- | --- | --- | --- | --- | --- | --- | --- | --- | --- | --- | --- | --- | --- | --- | --- | --- |
|  | B | SE | | *p* | *β* |  | B | SE | *p* | *β* | | |  | B | SE | *p* | *β* |  |
| Correlations |  |  | |  |  |  |  |  |  |  | | |  |  |  |  |  |  |
| Between-Person | -0.621 | 0.171 | | 0.000 | -0.235 |  | -0.597 | 0.226 | 0.008 | -0.261 | | |  | -0.850 | 0.321 | 0.008 | -0.291 |  |
| T1 | -0.151 | 0.138 | | 0.271 | -0.090 |  | 0.041 | 0.143 | 0.774 | 0.025 | | |  | -0.255 | 0.228 | 0.263 | -0.103 |  |
| Cross-Lagged Effects |  |  | |  |  |  |  |  |  |  | | |  |  |  |  |  |  |
| Problems 1 🡪 Comm. 2 | 0.153 | 0.966 | | 0.874 | 0.021 |  | 0.920 | 1.469 | 0.531 | 0.121 | | |  | 0.499 | 0.937 | 0.594 | 0.100 |  |
| Problems 2 🡪 Comm. 3 | 0.153 | 0.966 | | 0.874 | 0.011 |  | 0.920 | 1.469 | 0.531 | 0.072 | | |  | 0.499 | 0.937 | 0.594 | 0.087 |  |
| Comm. 1 🡪 Problems 2 | -0.000 | 0.012 | | 0.984 | -0.003 |  | -0.001 | 0.015 | 0.964 | -0.009 | | |  | 0.009 | 0.022 | 0.683 | 0.053 |  |
| Comm. 2 🡪 Problems 3 | -0.000 | 0.012 | | 0.984 | -0.002 |  | -0.001 | 0.015 | 0.964 | -0.006 | | |  | 0.009 | 0.022 | 0.683 | 0.049 |  |
| Stability Paths |  |  | |  |  |  |  |  |  |  | | |  |  |  |  |  |  |
| Comm. 1 🡪 Comm. 2 | -0.016 | 0.166 | | 0.924 | -0.019 |  | -0.015 | 0.179 | 0.933 | -0.018 | | |  | -0.025 | 0.162 | 0.879 | -0.030 |  |
| Comm. 2 🡪 Comm. 3 | -0.016 | 0.166 | | 0.924 | -0.013 |  | -0.015 | 0.179 | 0.933 | -0.013 | | |  | -0.025 | 0.162 | 0.879 | -0.021 |  |
| Problems 1 🡪 Problems 2 | -0.156 | 0.111 | | 0.159 | -0.248 |  | 0.023 | 0.210 | 0.912 | 0.057 | | |  | 0.325 | 0.163 | 0.046 | 0.311 |  |
| Problems 2 🡪 Problems 3 | -0.156 | 0.111 | | 0.159 | -0.100 |  | 0.023 | 0.210 | 0.912 | 0.033 | | |  | 0.325 | 0.163 | 0.046 | 0.364 |  |
| Correlated Change |  |  | |  |  |  |  |  |  |  | | |  |  |  |  |  |  |
| T2 | 0.108 | 0.259 | | 0.676 | 0.129 |  | 0.031 | 0.329 | 0.924 | 0.033 | | |  | -0.000 | 0.444 | 0.999 | -0.000 |  |
| T3 | -0.130 | 0.148 | | 0.381 | -0.080 |  | -0.102 | 0.119 | 0.391 | -0.070 | | |  | -0.053 | 0.210 | 0.802 | -0.025 |  |

*Note.* Problems: Denotes the Internalizing and Externalizing Problems, as specified in the columns; Comm.: Family Communication.

*Table S9*

Parameter Estimates from the Alternative Models for Family Flexibility that Fit Better Compared to the Fixed RICLPMs, controlling for adolescent sex and family socioeconomic status.

| Family Flexibility | Depressive symptoms – CLPM | | | |  | Depressive symptoms– RICLPM-free | | | |  | Anxiety – RICLPM-free | | | | |
| --- | --- | --- | --- | --- | --- | --- | --- | --- | --- | --- | --- | --- | --- | --- | --- |
|  | B | SE | *p* | *β* |  | B | SE | *p* | *β* |  | B | SE | *p* | *β* |  |
| Correlations |  |  |  |  |  |  |  |  |  |  |  |  |  |  |  |
| Between-Person | - | - | - | - |  | -0.240 | 0.131 | 0.067 | -0.131 |  | -0.212 | 0.146 | 0.149 | -0.139 |  |
| T1 | -0.146 | 0.125 | 0.243 | -0.051 |  | 0.057 | 0.117 | 0.623 | 0.057 |  | -0.015 | 0.124 | 0.902 | -0.015 |  |
| Cross-Lagged Effects |  |  |  |  |  |  |  |  |  |  |  |  |  |  |  |
| Problems 1 🡪 Flexibility 2 | -0.662 | 0.213 | 0.002 | -0.104 |  | -0.115 | 0.940 | 0.902 | -0.017 |  | 0.794 | 0.942 | 0.399 | 0.120 |  |
| Problems 2 🡪 Flexibility 3 | -0.662 | 0.213 | 0.002 | -0.094 |  | -0.297 | 1.159 | 0.798 | -0.036 |  | -1.156 | 1.077 | 0.283 | -0.156 |  |
| Flexibility 1 🡪 Problems 2 | 0.002 | 0.005 | 0.666 | 0.014 |  | 0.041 | 0.025 | 0.103 | 0.315 |  | 0.007 | 0.024 | 0.770 | 0.047 |  |
| Flexibility 2 🡪 Problems 3 | 0.002 | 0.005 | 0.666 | 0.012 |  | -0.018 | 0.016 | 0.241 | -0.108 |  | -0.013 | 0.014 | 0.333 | -0.081 |  |
| Stability Paths |  |  |  |  |  |  |  |  |  |  |  |  |  |  |  |
| Flexibility 1 🡪 Flexibility 2 | 0.471 | 0.047 | 0.000 | 0.481 |  | -0.201 | 0.169 | 0.235 | -0.191 |  | -0.213 | 0.159 | 0.180 | -0.204 |  |
| Flexibility 2 🡪 Flexibility 3 | 0.471 | 0.047 | 0.000 | 0.441 |  | -0.073 | 0.133 | 0.584 | -0.070 |  | -0.098 | 0.135 | 0.470 | -0.094 |  |
| Problems 1 🡪 Problems 2 | 0.582 | 0.036 | 0.000 | 0.606 |  | -0.229 | 0.196 | 0.243 | -0.268 |  | 0.084 | 0.177 | 0.636 | 0.090 |  |
| Problems 2 🡪 Problems 3 | 0.582 | 0.036 | 0.000 | 0.528 |  | 0.285 | 0.156 | 0.068 | 0.208 |  | 0.452 | 0.131 | 0.001 | 0.385 |  |
| Correlated Change |  |  |  |  |  |  |  |  |  |  |  |  |  |  |  |
| T2 | 0.014 | 0.069 | 0.846 | 0.007 |  | 0.101 | 0.189 | 0.594 | 0.124 |  | -0.058 | 0.174 | 0.740 | -0.059 |  |
| T3 | 0.014 | 0.069 | 0.846 | 0.007 |  | -0.042 | 0.102 | 0.682 | -0.034 |  | -0.104 | 0.097 | 0.283 | -0.094 |  |

*Note*: CLPM: Cross-Lagged Panel Model; RICLPM-free: Random-Intercept Cross-Lagged Panel Model with no time-invariance constrains; Problems: Internalizing/Externalizing Problems, as specified in the columns; Flexibility: Family Flexibility.

*Table S10*

Parameter Estimates from the Alternative Models for Family Cohesion that Fit Better Compared to the Fixed RICLPMs, controlling for adolescent sex and family socioeconomic status.

| Family Cohesion | Depressive symptoms – CLPM | | | |  | Anxiety – CLPM | | | |  | Anxiety – RICLPM-free | | | |  | Anger – CLPM | | | |
| --- | --- | --- | --- | --- | --- | --- | --- | --- | --- | --- | --- | --- | --- | --- | --- | --- | --- | --- | --- |
|  | B | SE | p | β |  | B | SE | p | β |  | B | SE | p | β |  | B | SE | p | β |
| Correlations |  |  |  |  |  |  |  |  |  |  |  |  |  |  |  |  |  |  |  |
| Between-Person | - | - | - | - |  | - | - | - | - |  | -0.286 | 0.175 | 0.102 | -0.187 |  | - | - | - | - |
| T1 | -0.249 | 0.156 | 0.112 | -0.080 |  | -0.293 | 0.141 | 0.038 | -0.105 |  | -0.026 | 0.151 | 0.864 | -0.020 |  | -0.473 | 0.238 | 0.047 | -0.116 |
| Cross-Lagged Effects |  |  |  |  |  |  |  |  |  |  |  |  |  |  |  |  |  |  |  |
| Problems 1 🡪 Cohesion 2 | -0.610 | 0.219 | 0.005 | -0.091 |  | -0.532 | 0.259 | 0.040 | -0.073 |  | 0.231 | 1.025 | 0.822 | 0.032 |  | -0.536 | 0.161 | 0.001 | -0.104 |
| Problems 2 🡪 Cohesion 3 | -0.610 | 0.219 | 0.005 | -0.083 |  | -0.532 | 0.259 | 0.040 | -0.068 |  | -0.880 | 1.077 | 0.414 | -0.113 |  | -0.536 | 0.161 | 0.001 | -0.099 |
| Cohesion 1 🡪 Problems 2 | 0.003 | 0.005 | 0.573 | 0.019 |  | -0.002 | 0.005 | 0.635 | -0.017 |  | 0.001 | 0.018 | 0.946 | 0.010 |  | 0.003 | 0.007 | 0.642 | 0.018 |
| Cohesion 2 🡪 Problems 3 | 0.003 | 0.005 | 0.573 | 0.016 |  | -0.002 | 0.005 | 0.635 | -0.016 |  | -0.012 | 0.016 | 0.467 | -0.078 |  | 0.003 | 0.007 | 0.642 | 0.017 |
| Stability Paths |  |  |  |  |  |  |  |  |  |  |  |  |  |  |  |  |  |  |  |
| Cohesion 1 🡪 Cohesion 2 | 0.549 | 0.041 | 0.000 | 0.580 |  | 0.549 | 0.040 | 0.000 | 0.578 |  | 0.097 | 0.143 | 0.500 | 0.102 |  | 0.547 | 0.039 | 0.000 | 0.575 |
| Cohesion 2 🡪 Cohesion 3 | 0.549 | 0.041 | 0.000 | 0.522 |  | 0.549 | 0.040 | 0.000 | 0.525 |  | 0.112 | 0.143 | 0.436 | 0.112 |  | 0.547 | 0.039 | 0.000 | 0.524 |
| Problems 1 🡪 Problems 2 | 0.584 | 0.036 | 0.000 | 0.609 |  | 0.606 | 0.041 | 0.000 | 0.621 |  | 0.096 | 0.187 | 0.608 | 0.102 |  | 0.574 | 0.039 | 0.000 | 0.581 |
| Problems 2 🡪 Problems 3 | 0.584 | 0.036 | 0.000 | 0.528 |  | 0.606 | 0.041 | 0.000 | 0.580 |  | 0.456 | 0.148 | 0.002 | 0.389 |  | 0.574 | 0.039 | 0.000 | 0.579 |
| Correlated Change |  |  |  |  |  |  |  |  |  |  |  |  |  |  |  |  |  |  |  |
| T2 | -0.058 | 0.073 | 0.432 | -0.032 |  | -0.123 | 0.065 | 0.059 | -0.074 |  | -0.188 | 0.165 | 0.254 | -0.169 |  | -0.068 | 0.088 | 0.443 | -0.026 |
| T3 | -0.058 | 0.073 | 0.432 | -0.032 |  | -0.123 | 0.065 | 0.059 | -0.078 |  | -0.078 | 0.099 | 0.433 | -0.066 |  | -0.068 | 0.088 | 0.443 | -0.031 |

*Note:* CLPM: Cross-Lagged Panel Model; RICLPM-free: Random-Intercept Cross-Lagged Panel Model with no time-invariance constrains; Problems: Internalizing/Externalizing Problems, as specified in the columns; Cohesion: Family Cohesion.

*Table S11*

Parameter Estimates from the Alternative Models for Family Communication that Fit Better Compared to the Fixed RICLPMs, controlling for adolescent sex and family socioeconomic status.

| Family Communication | Depressive symptoms – CLPM | | | |  | Anxiety – CLPM | | | |  | Anxiety – RICLPM-free | | | |  | Anger – CLPM | | | |
| --- | --- | --- | --- | --- | --- | --- | --- | --- | --- | --- | --- | --- | --- | --- | --- | --- | --- | --- | --- |
|  | B | SE | *p* | *β* |  | B | SE | *p* | *β* |  | B | SE | *p* | *β* |  | B | SE | *p* | *β* |
| Correlations |  |  |  |  |  |  |  |  |  |  |  |  |  |  |  |  |  |  |  |
| Between-Person | - | - | - | - |  | - | - | - | - |  | -0.566 | 0.206 | 0.006 | -0.274 |  | - | - | - | - |
| T1 | -0,646 | 0,209 | 0,002 | -0,163 |  | -0.417 | 0.192 | 0.030 | -0.116 |  | 0.039 | 0.200 | 0.845 | 0.026 |  | -0.897 | 0.273 | 0.001 | -0.172 |
| Cross-Lagged Effects |  |  |  |  |  |  |  |  |  |  |  |  |  |  |  |  |  |  |  |
| Problems 1 🡪 Comm. 2 | -0,831 | 0,272 | 0,002 | -0,096 |  | -0.317 | 0.307 | 0.302 | -0.034 |  | 1.798 | 1.400 | 0.199 | 0.219 |  | -0.266 | 0.239 | 0.266 | -0.040 |
| Problems 2 🡪 Comm. 3 | -0,831 | 0,272 | 0,002 | -0,084 |  | -0.317 | 0.307 | 0.302 | -0.026 |  | -0.569 | 1.388 | 0.682 | -0.054 |  | -0.266 | 0.239 | 0.266 | -0.036 |
| Comm. 1 🡪 Problems 2 | -0,003 | 0,004 | 0,516 | -0,023 |  | -0.007 | 0.004 | 0.067 | -0.068 |  | -0.016 | 0.016 | 0.318 | -0.158 |  | -0.003 | 0.005 | 0.547 | -0.022 |
| Comm. 2 🡪 Problems 3 | -0,003 | 0,004 | 0,516 | -0,021 |  | -0.007 | 0.004 | 0.067 | -0.063 |  | 0.011 | 0.012 | 0.368 | 0.083 |  | -0.003 | 0.005 | 0.547 | -0.021 |
| Stability Paths |  |  |  |  |  |  |  |  |  |  |  |  |  |  |  |  |  |  |  |
| Comm. 1 🡪 Comm. 2 | 0,572 | 0,041 | 0,000 | 0,594 |  | 0.581 | 0.040 | 0.000 | 0.605 |  | -0.078 | 0.152 | 0.609 | -0.085 |  | 0.580 | 0.040 | 0.000 | 0.601 |
| Comm. 2 🡪 Comm. 3 | 0,572 | 0,041 | 0,000 | 0,523 |  | 0.581 | 0.040 | 0.000 | 0.530 |  | 0.168 | 0.192 | 0.382 | 0.139 |  | 0.580 | 0.040 | 0.000 | 0.530 |
| Problems 1 🡪 Problems 2 | 0,580 | 0,037 | 0,000 | 0,603 |  | 0.599 | 0.040 | 0.000 | 0.615 |  | 0.114 | 0.169 | 0.500 | 0.122 |  | 0.576 | 0.040 | 0.000 | 0.581 |
| Problems 2 🡪 Problems 3 | 0,580 | 0,037 | 0,000 | 0,527 |  | 0.599 | 0.040 | 0.000 | 0.574 |  | 0.485 | 0.122 | 0.000 | 0.413 |  | 0.576 | 0.040 | 0.000 | 0.582 |
| Correlated Change |  |  |  |  |  |  |  |  |  |  |  |  |  |  |  |  |  |  |  |
| T2 | -0,085 | 0,087 | 0,331 | -0,037 |  | -0.140 | 0.082 | 0.086 | -0.066 |  | -0.056 | 0.225 | 0.803 | -0.046 |  | -0.237 | 0.128 | 0.064 | -0.072 |
| T3 | -0,085 | 0,087 | 0,331 | -0,036 |  | -0.140 | 0.082 | 0.086 | -0.067 |  | -0.122 | 0.116 | 0.290 | -0.074 |  | -0.237 | 0.128 | 0.064 | -0.080 |

*Note:* CLPM: Cross-Lagged Panel Model; RICLPM-free: Random-Intercept Cross-Lagged Panel Model with no time-invariance constrains; Problems: Internalizing/Externalizing Problems, as specified in the columns; Comm.: Family Communication.

*Table S12*

| Family Communication | Anger – RICLPM-free | | | | |
| --- | --- | --- | --- | --- | --- |
|  | B | SE | *p* | *β* |  |
| Correlations |  |  |  |  |  |
| Between-Person | -0.775 | 0.286 | 0.007 | -0.261 |  |
| T1 | -0.247 | 0.288 | 0.390 | -0.110 |  |
| Cross-Lagged Effects |  |  |  |  |  |
| Problems 1 🡪 Comm. 2 | 0.708 | 0.930 | 0.447 | 0.128 |  |
| Problems 2 🡪 Comm. 3 | -0.161 | 0.939 | 0.864 | -0.025 |  |
| Comm. 1 🡪 Problems 2 | -0.011 | 0.027 | 0.670 | -0.068 |  |
| Comm. 2 🡪 Problems 3 | 0.013 | 0.018 | 0.473 | 0.072 |  |
| Stability Paths |  |  |  |  |  |
| Comm. 1 🡪 Comm. 2 | -0.065 | 0.157 | 0.680 | -0.071 |  |
| Comm. 2 🡪 Comm. 3 | 0.152 | 0.211 | 0.470 | 0.127 |  |
| Problems 1 🡪 Problems 2 | 0.168 | 0.182 | 0.357 | 0.164 |  |
| Problems 2 🡪 Problems 3 | 0.381 | 0.122 | 0.002 | 0.391 |  |
| Correlated Change |  |  |  |  |  |
| T2 | -0.164 | 0.333 | 0.622 | -0.081 |  |
| T3 | -0.106 | 0.203 | 0.602 | -0.047 |  |

Parameter Estimates from the Alternative Model for Family Communication and Anger, which Fit Better Compared to the Fixed RICLPMs, controlling for adolescent sex and family socioeconomic status.

*Note:* RICLPM-free: Random-Intercept Cross-Lagged Panel Model with no time-invariance constrains; Problems: Internalizing/Externalizing Problems, as specified in the columns; Comm.: Family Communication.
